# N6-methyladenosine demethylase FTO impairs hepatic ischemia–reperfusion injury via inhibiting Drp1-mediated mitochondrial fragmentation

**DOI:** 10.1038/s41419-021-03622-x

**Published:** 2021-05-04

**Authors:** Ying Dong Du, Wen Yuan Guo, Cong Hui Han, Ying Wang, Xiao Song Chen, Da Wei Li, Jin Long Liu, Ming Zhang, Nan Zhu, Xin Wang

**Affiliations:** 1 Department of Transplantation and Hepatic Surgery, 970 Hospital of the PLA Joint Logistic Support Force, Yantai, China; 2 Department of Liver Surgery and Organ Transplantation, Changzheng Hospital, Naval Medical University, Shanghai, China; 3 Department of Urology, The Affiliated School of Clinical Medicine of Xuzhou Medical College, Xuzhou Central Hospital, Xuzhou, China; 4 Department of Transplantation and Hepatic Surgery, Ren Ji Hospital, Shanghai Jiaotong University School of Medicine, Shanghai, China; 5 Department of Urology, Ren Ji Hospital, Shanghai Jiaotong University School of Medicine, Shanghai, China; 6 Department of Biotechnology and Pathology, School of Medical Technology, Shanghai University of Medicine & Health Sciences, Shanghai, China; 7 The Fifth Hepatic Surgery Department, Shanghai Eastern Hepatobiliary Surgery Hospital, Shanghai, China; 8 Department of Hepatic Surgery, Zhongshan Hospital, Fudan University, Shanghai, China

**Keywords:** Mechanisms of disease, RNA modification

## Abstract

Despite N6-methyladenosine (m6A) is functionally important in various biological processes, its role and the underlying regulatory mechanism in the liver remain largely unexplored. In the present study, we showed that fat mass and obesity-associated protein (FTO, an m6A demethylase) was involved in mitochondrial function during hepatic ischemia–reperfusion injury (HIRI). We found that the expression of m6A demethylase FTO was decreased during HIRI. In contrast, the level of m6A methylated RNA was enhanced. Adeno-associated virus-mediated liver-specific overexpression of FTO (AAV8-TBG-FTO) ameliorated the HIRI, repressed the elevated level of m6A methylated RNA, and alleviated liver oxidative stress and mitochondrial fragmentation in vivo and in vitro. Moreover, dynamin-related protein 1 (Drp1) was a downstream target of FTO in the progression of HIRI. FTO contributed to the hepatic protective effect via demethylating the mRNA of Drp1 and impairing the Drp1-mediated mitochondrial fragmentation. Collectively, our findings demonstrated the functional importance of FTO-dependent hepatic m6A methylation during HIRI and provided valuable insights into the therapeutic mechanisms of FTO.

## Introduction

Hepatic ischemia–reperfusion injury (HIRI) is a complication observed during liver resection and transplantation^1^. Inevitable HIRI mostly occurs with transplantation surgery during harvest, organ resection, and graft implant. It can also be accompanied by hemorrhagic shock and trauma^2,3^. Up to 10% of acute graft dysfunction exists in liver transplantation, although the surgical techniques and allograft preservation have been greatly improved^4^. This persistent damage to transplanted organs leads to significant graft loss in the first year after transplantation, resulting in the necessity of repeat transplantation and contributing to a serious shortage of donor organs available for transplantation^5^. Therefore, a good understanding of the underlying mechanism and effective intervention measures to minimize the adverse reactions of HIRI can significantly increase the cure rate of liver transplant recipients and extend their lifespan.

Various epigenetic studies have focused on histone modifications, DNA methylation, and chromatin remodeling. Similarly, coding RNAs have a series of covalent modifications that manage gene expression by affecting RNA stability and translation^6^. As a type of most abundant mRNA modification, N6-methyladenosine (m6A) is transcriptome-wide presented in most RNAs and normally enriched near the 5′ UTRs^7–9^. Emerging evidence shows that mammalian m6A is dynamically regulated and involved in various biological progress^10,11^. Especially, m6A has been reported to be involved in several IRIs, including myocardial^12^, renal^13^, and neuronal^14–16^ IRIs. However, the pathological role and regulatory mechanism of this newly emerging RNA modification in the progression of HIRI have not been illustrated yet. Therefore, it is urgently necessary to unveil its particular role in HIRI to develop new therapeutic strategies for liver transplant recipients.

The reversible m6A RNA modification is coordinated by a methyltransferase (m6A “writers”), m6A reader proteins, and demethylase (m6A “erasers”). These members cover more than 13 enzymes. The m6A “writers” complex consists of METTL3, METTL14, WTAP, CBLL1, RBM15, ZC3H13, and VIRMA, which are responsible for the methylation of target RNA transcripts^11,17,18^. Moreover, m6A readers, including YTHDF1-3, YTHDC1, IGF2BPs, and eIF3, recognize these m6A modifications to direct RNA alternative splicing, translation, localization, and RNA stability among other processes^16^. However, as the m6A “erasers”, fat mass and obesity-associated protein (FTO) and ALKBH5 remove m6A from the aforementioned target transcripts^19–21^. FTO, the first identified m6A demethylase, belongs to the ALKB family of Fe (II)/α-ketoglutarate-dependent dioxygenases^12^. FTO mediates multiple RNA modifications, including m6A and m6Am in mRNA and snRNA as well as m1A in tRNA. Recent studies have demonstrated that the demethylase activity of FTO selectively demethylates cardiac contractile transcripts, preventing their degradation and improving their protein expressions in ischemic injury^12,22^. However, the underlying epigenetic mechanism of FTO in HIRI remains largely unexplored.

In the present study, we investigated the pathological role, the underlying mechanism of the m6A-methylated RNA level, and its demethylase FTO in the progression of HIRI. We found that the level of m6A-methylated RNA was gradually and significantly elevated at 3, 6, and 12 h after IRI in a murine HIRI model. Conversely, the expression of FTO was suppressed during IRI. Functionally, adeno-associated virus-mediated liver-specific overexpression of FTO (AAV-TBG-FTO) attenuated the cellular damage in hepatic cells in vitro and protected against IRI in a murine model in vivo. Moreover, the enhanced expression of FTO attenuated the IRI-induced mitochondrial fragmentation. Furthermore, we identified dynamin-related protein 1 (Drp1) as a downstream target of FTO in the progression of HIRI. Besides, liver-specific overexpression of Drp1 abrogated the protective effect of FTO against IRI. Collectively, these results demonstrated that the m6A demethylase FTO ameliorated HIRI via inhibiting Drp1-mediated mitochondrial fragmentation.

## Method and materials

### Animals

The experimental protocols for C57BL/6 mice (male, 6–8 weeks, Shanghai Slac Laboratory Animal Co., Ltd., China) were approved by the Institutional Animal Care and Use Committee of Ren Ji Hospital.

### Murine model of HIRI

The murine model of HIRI was established as previously described^23^. Briefly, male C57BL/6 mice were anesthetized by intraperitoneal injection of chloral hydrate (4 mL/kg), followed by midline laparotomy. Noninvasive arterial clamps were inserted between the left lobe and the base of the median hepatic lobe to block the blood supply. After 60 min of ischemia, the forceps were removed, and reperfusion was conducted. Mice were sacrificed at 3, 6, and 12 h after reperfusion.

### Serum aminotransferase activities

The serum levels of ALT and AST were determined to estimate the degree of hepatocyte damage after HIRI as previously described^23^ using a Hitachi 7600 automatic analyzer (Hitachi, Ltd., Tokyo, Japan).

### Histology

Histology was performed as previously described^23^.

### Global m6A measurements

The level of global m6A in total RNA was quantified by EpiQuik m6A RNA Methylation Quantification Kit (Epigentek Group, Farmingdale, NY) as previously described^24^.

### m6A dot blotting analysis

m6A dot blotting analysis was used to detect the qualitative m6A modifications as previously described^25^. Briefly, poly-A RNA was purified by Dynabeads mRNA Purification Kit (Thermo Fisher, Carlsbad, CA, USA) and spotted on an Amersham Hybond-XL membrane. After incubation with the anti-m6A primary antibody and the mouse-HRP secondary antibody, the membrane was incubated with Pierce ECL2 Western Blotting Substrate, and exposed to X-Ray Super RX Films.

### Hepatocyte isolation

Hepatocytes were isolated from mice and cultured as previously described^23^.

### Quantitative real-time PCR (qRT-PCR)

Briefly, 1 μg purified RNA was reversely transcribed into cDNA, and qRT-PCR was performed using the ABI ViiA 7 Real-Time PCR System (Applied Biosystems) as previously described^24^. GAPDH was selected as a housekeeping gene. The specific primer sequences were as follows:

**Table:** 

**Gene name**	**Forward primer (5ʹ-3ʹ)**	**Reverse primer (5ʹ-3ʹ)**
Mouse FTO	GGCGGCTTTAGTAGCAGCAT	CCAAGTGTCTTCAAGCTCCTC
Mouse Drp1	TCAGATCGTCGTAGTGGGAA	TCTTCTGGTGAAACGTGGAC
Mouse GAPDH	AATCCCATCACCATCTTCCAG	AAATGAGCCCCAGCCTTC

### Western blotting analysis

The western blotting analysis was performed as previously described^23^. Briefly, total proteins were isolated from liver tissues or hepatocytes, separated by 12% sodium dodecyl sulfate polyacrylamide gel electrophoresis, and transferred onto polyvinylidene difluoride membranes. Membranes were then incubated with the primary antibodies as follows: anti-FTO (Abcam, Cambridge, UK), anti-Drp1 (Abcam), and anti-GAPDH (Santa Cruz Biotechnology).

### Assays for glutathione (GSH), glutathione peroxidase (GSH-Px), superoxide dismutase (SOD), and malondialdehyde (MDA) in liver tissue

The activities of GSH, GSH-Px, SOD, and MDA in liver tissues were determined using SOD, GSH, GSH-Px, MDA, and MPO detection kits (Simo Biomedical Technology, Shanghai, China), respectively, following the manufacturer’s instructions.

### Electron microscopy

Liver tissue samples were fixed in 2.5% glutaraldehyde solution, followed by dehydration, embedding, and sectioning. Finally, tissue sections were photographed using a Hitachi 7500 transmission electron microscope (Hitachi, Tokyo, Japan).

### Detection of mitochondrial fragmentation

After incubation with MitoTracker Red (75 nmol/L, 20 min, Thermo Fisher, Carlsbad, CA, USA), primary hepatocytes were labeled with MitoTracker Red CMXRos (Thermo Fisher, Carlsbad, CA, USA) and photographed using an LSM900 Zeiss confocal laser scanning microscope.

### Cell viability assay

The cell viability of primary hepatocytes was detected by Cell Counting Kit-8 (Simo Biomedical Technology, Shanghai, China) as previously described^23^.

### Me-RIP assay

The methylated m6A RNA immunoprecipitation (me-RIP) was performed to analyze the level of methylated Drp1 mRNA using the anti-m^6^A antibody (Abcam, ab151230) as previously described^24^.

### Statistical analysis

Data were expressed as means ± SEM. A two-tailed *t* test (unpaired) was used for comparisons between two groups. ANOVA followed by the post hoc Bonferroni test was adopted for multiple comparisons using GraphPad Prism^®^ version 6.0 software (GraphPad Software, Inc., La Jolla, CA, USA). A *p* value less than 0.05 was considered statistically significant.

## Results

### The expression of m6A demethylase FTO is decreased in the liver tissue upon IRI

**Fig. 1:**
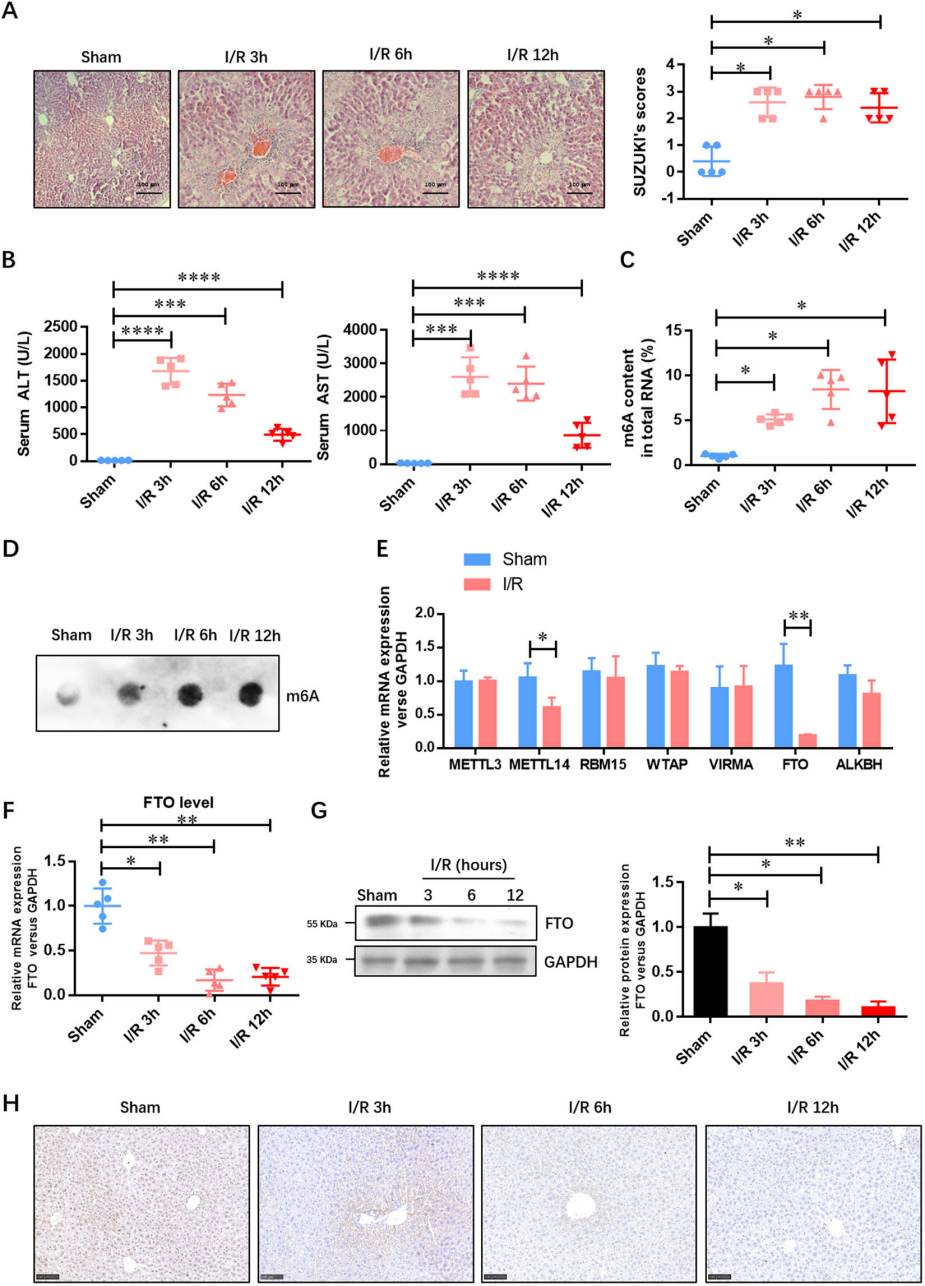
The expression of m6A demethylase FTO is decreased by HIRI. **A** Representative histopathologic images of liver sections harvested at 24 h after reperfusion (H&E staining; scale bars, 100 μm). The damage degree was graded using Suzuki criteria. **B** Blood samples were collected to determine the ALT and AST levels (U/L). **C** The total level of methylated RNA (m6A) in hepatic tissues was detected by EpiQuik m6A RNA Methylation Quantification Kit. **D** The total level of methylated RNA (m6A) in hepatic tissues was detected by dot blotting analysis. **E** The expressions of major m6A methyltransferases (METTL3, METTL14, RBM15, WTAP, and VIRMA) and demethylases (FTO and ALKBH5) at the mRNA level were examined by qRT-PCR. **F** The expression of FTO at the mRNA level was examined by qRT-PCR. **G** The expression of FTO at the protein level was verified by Western blotting analysis. **H** The expression of FTO at the protein level was verified by IHC. *N* = 5; *P < 0.05 and **P < 0.01, ***P < 0.001 vs. sham group.

To evaluate the role of m6A methylated RNA in IRI, we established a murine model of HIRI. The liver histopathological changes in the IRI group were confirmed. Figure 1A shows that there was extensive necrosis in IRI liver samples accompanied by sinusoidal congestion and cell swelling, which was never appeared in the sham group. The damage degree was also evaluated by blinded pathologists based on Suzuki criterion scores, and the results suggested a higher score in the IRI group compared with the sham group (Fig. 1A). At 3, 6, and 12 h after the IRI procedure, the levels of serum ALT and AST (two indices of hepatocellular injury) were also significantly increased in the IRI group (Fig. 1B). The level of m6A methylated RNA was consistently induced at 3, 6, and 12 h after IRI (Fig. 1C, D). We then analyzed the expressions of major m6A methyltransferases (METTL3, METTL14, RBM15, WTAP, and VIRMA) and demethylases (FTO and ALKBH5) at the mRNA level in the liver tissue of the sham group and 12-h IRI group. Figure 1E shows that the expressions of METTL14 and FTO were decreased in the IRI group, which was opposite with the m6A accumulation. Therefore, we hypothesized that FTO downregulation contributed to the m6A accumulation in the liver tissue of the IRI group. The downregulation of FTO at both the mRNA and protein levels at 3, 6, and 12 h after IRI was further verified by qRT-PCR (Fig. 1F), Western blotting analysis (Fig. 1G), and immunohistochemistry (IHC) (Fig. 1H). These results indicated that FTO-mediated m6A demethylation might contribute to the development of HIRI.

### Adeno-associated virus-mediated liver-specific overexpression of FTO (AAV8-TBG-FTO) attenuates the HIRI and represses the elevated level of m6A methylated RNA in vivo

**Fig. 2:**
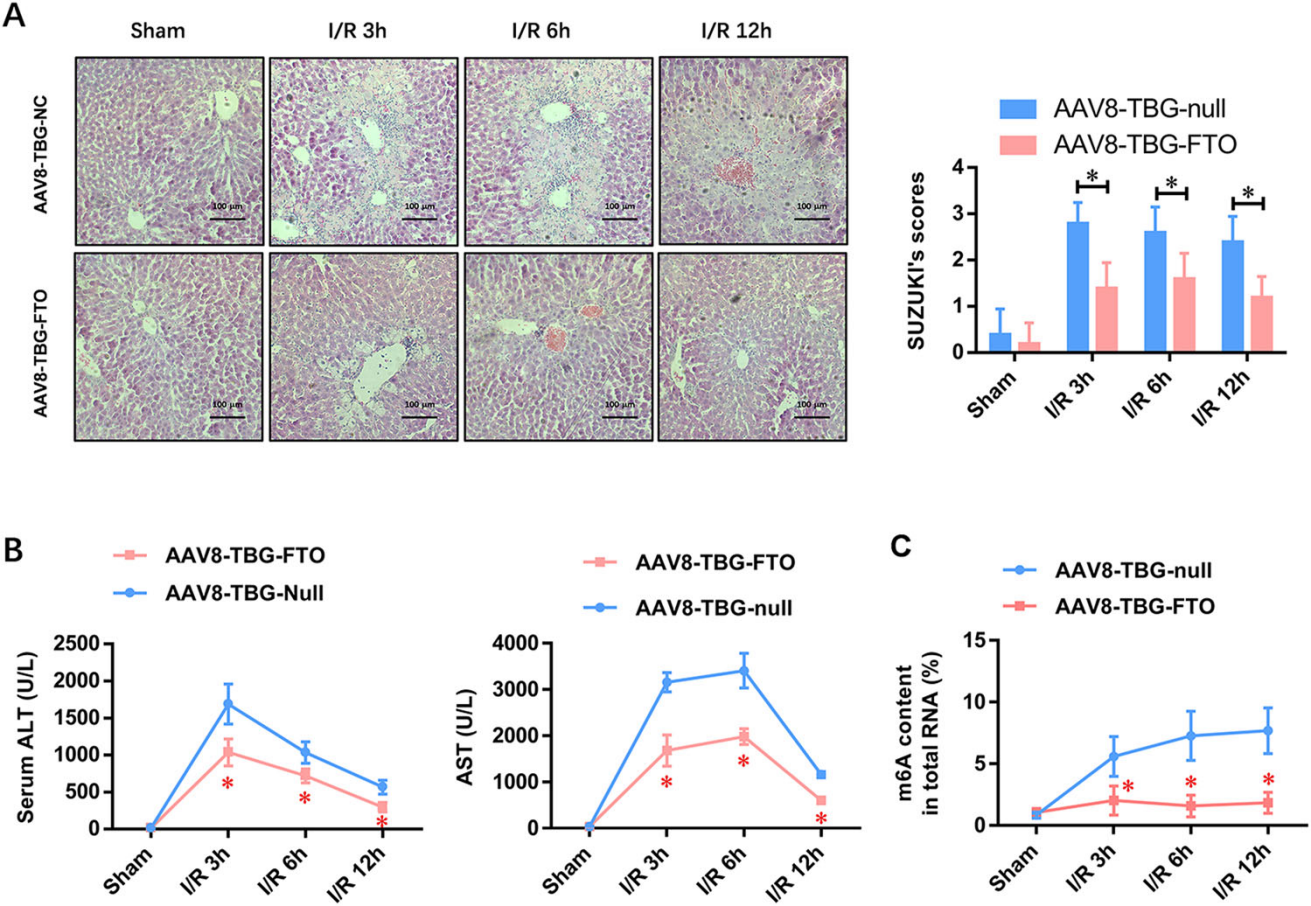
Adeno-associated virus-mediated liver-specific overexpression of FTO (AAV8-TBG-FTO) attenuates the HIRI and represses the elevated level of m6A methylated RNA in vivo. **A** Representative histopathologic images of liver sections harvested at 24 h after reperfusion (H&E staining; scale bars, 100 μm). The damage degree was graded using Suzuki criteria. **B** Blood samples were collected to determine the ALT and AST levels (U/L). **C** The total level of methylated RNA (m6A) in hepatic tissues was detected by EpiQuik m6A RNA Methylation Quantification Kit. *N* = 5; *P < 0.05 vs. AAV8-TBG-NC group.

To further explore the potential role of FTO-mediated m6A demethylation in the progression of HIRI, an adeno-associated virus serotype 8 (AAV8) was adopted to hepatocyte-specifically express FTO recombinase under the trigger of the thyroxin-binding globulin (TBG) promoter (AAV8-TBG-FTO). Compared with the vector control group (AAV8-TBG-null), the liver histopathological changes, including the area of necrosis, sinusoidal congestion, and cell swelling, were dramatically attenuated, evidenced by the reduced Suzuki criterion scores (Fig. 2A). Moreover, the levels of serum ALT and AST were also significantly reduced in the AAV8-TBG-FTO group at 3, 6, and 12 h after IRI (Fig. 2B), whereas the IRI-elevated m6A methylated RNA level was reversed and became stable at 3, 6, and 12 h after IRI (Fig. 2C). The results indicated that FTO played a critical role in HIRI.

### Liver-specific overexpression of FTO alleviates liver oxidative stress and mitochondrial fragmentation in vivo

**Fig. 3:**
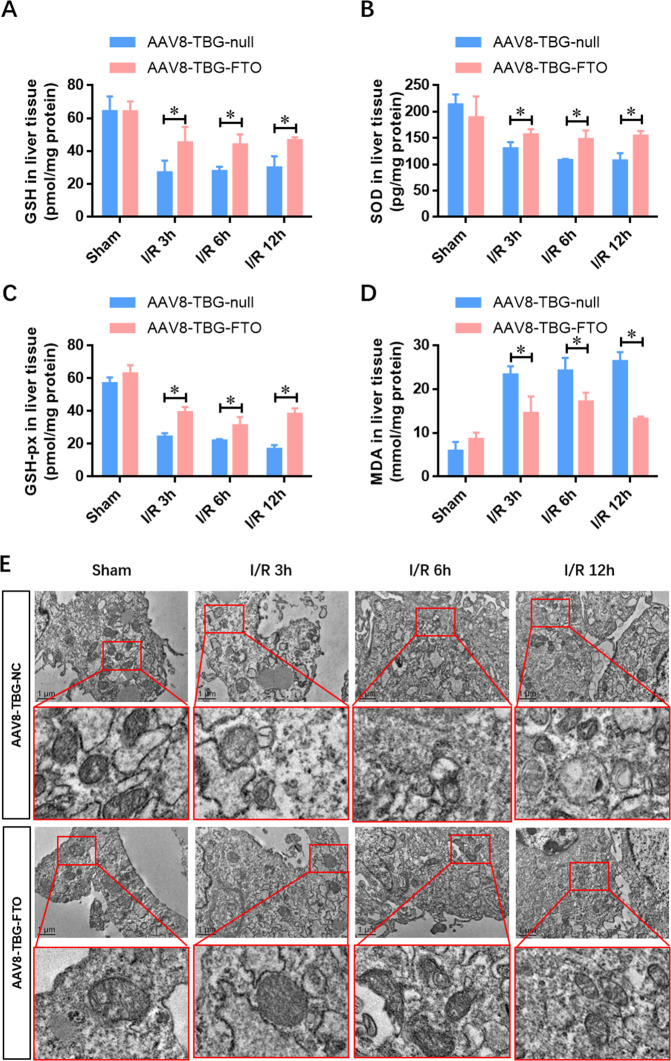
Liver-specific overexpression of FTO alleviates liver oxidative stress and mitochondrial fragmentation in vivo. **A**–**D** Tissue levels of GSH, GSH-Px, SOD, and MDA were detected using ELISA at indicated time points after reperfusion. **E** Representative electron microscope photos of the mitochondria in the hepatic tissues infected with control AAV8-TBG-null and AAV8-TBG-FTO during IRI. *N* = 5; *P < 0.05 vs. AAV8-TBG-NC group.

To explore the effect of FTO on oxidative stress during IRI, we determined the activities of oxidative relative enzymes (GSH, GSHPx, SOD, and MDA). The levels of GSH, GSH-Px, and SOD were dramatically decreased in liver cells after IRI compared with the sham group. FTO overexpression (AAV8-TBG-FTO) significantly increased the activities of GSH, GSH-Px, and SOD compared with the vector control group (AAV8-TBG-null) after IRI (Fig. 3A–C). Accordingly, the increased MDA activity after IRI was attenuated by FTO overexpression (Fig. 3D). Taken together, these results indicated that liver-specific overexpression of FTO ameliorated the oxidative stress in HIRI.

The mitochondrial dysfunction and fragmentation have been implicated in the induction of oxidative stress, which greatly contributes to the pathogenesis of several IRIs. To explore the effect of FTO overexpression on mitochondria in HIRI, electron microscopy was adopted to assess the fragmented mitochondria during IRI (Fig. 3E). Notably, the restoration of FTO diminished the number of fragmented mitochondria in HIRI (Fig. 3E). These results indicated that FTO improved the mitochondrial quality and alleviated the oxidative stress in the hepatocytes upon IRI.

### FTO overexpression alleviates the IRI-impaired cell viability and attenuates mitochondrial fragmentation

**Fig. 4:**
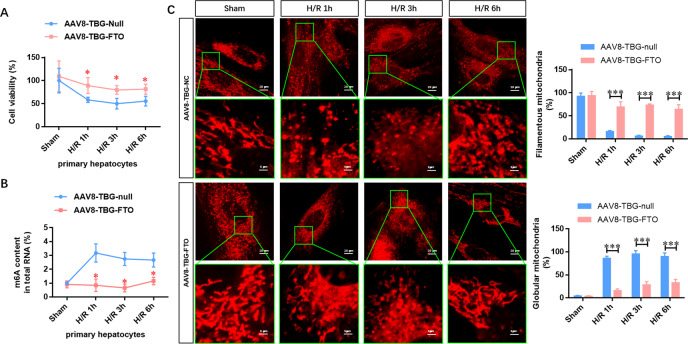
FTO overexpression alleviates the IRI-impaired cell viability and attenuates mitochondrial fragmentation. **A** Cell viability in the hepatocytes infected with control AAV8-TBG-null and AAV8-TBG-FTO during IRI was detected by CCK-8 assays. **B** The total level of methylated RNA (m6A) in hepatocytes was detected by EpiQuik m6A RNA Methylation Quantification Kit. **C** Confocal microscopy was used to analyze the elongated, intermediate, fragmented mitochondria of cardiomyocytes infected with control AAV8-TBG-null and AAV8-TBG-FTO during IRI. *N* = 3; *P < 0.05 and ***P < 0.001 vs. AAV8-TBG-NC group.

To further confirm the role of FTO-mediated m6A demethylation in the progression of HIRI, FTO was overexpressed in the isolated primary hepatocytes, followed by IRI. Figure 4A shows that the cell viability was gradually decreased after IRI compared with the sham group, whereas these reductions were diminished by FTO overexpression. Moreover, the elevated m6A methylated RNA level during IRI was disappeared in FTO-overexpressing primary hepatocytes (Fig. 4B). Next, confocal laser scanning microscopy showed that FTO overexpression increased the ratio of filamentous mitochondria and decreased globular mitochondria during IRI (Fig. 4C). These data further suggested that FTO improved the mitochondrial quality and alleviated the damage in hepatocytes upon IRI.

### Liver-specific FTO overexpression suppresses the IRI-induced expression of Drp1 in hepatocytes upon IRI in vivo and in vitro

**Fig. 5:**
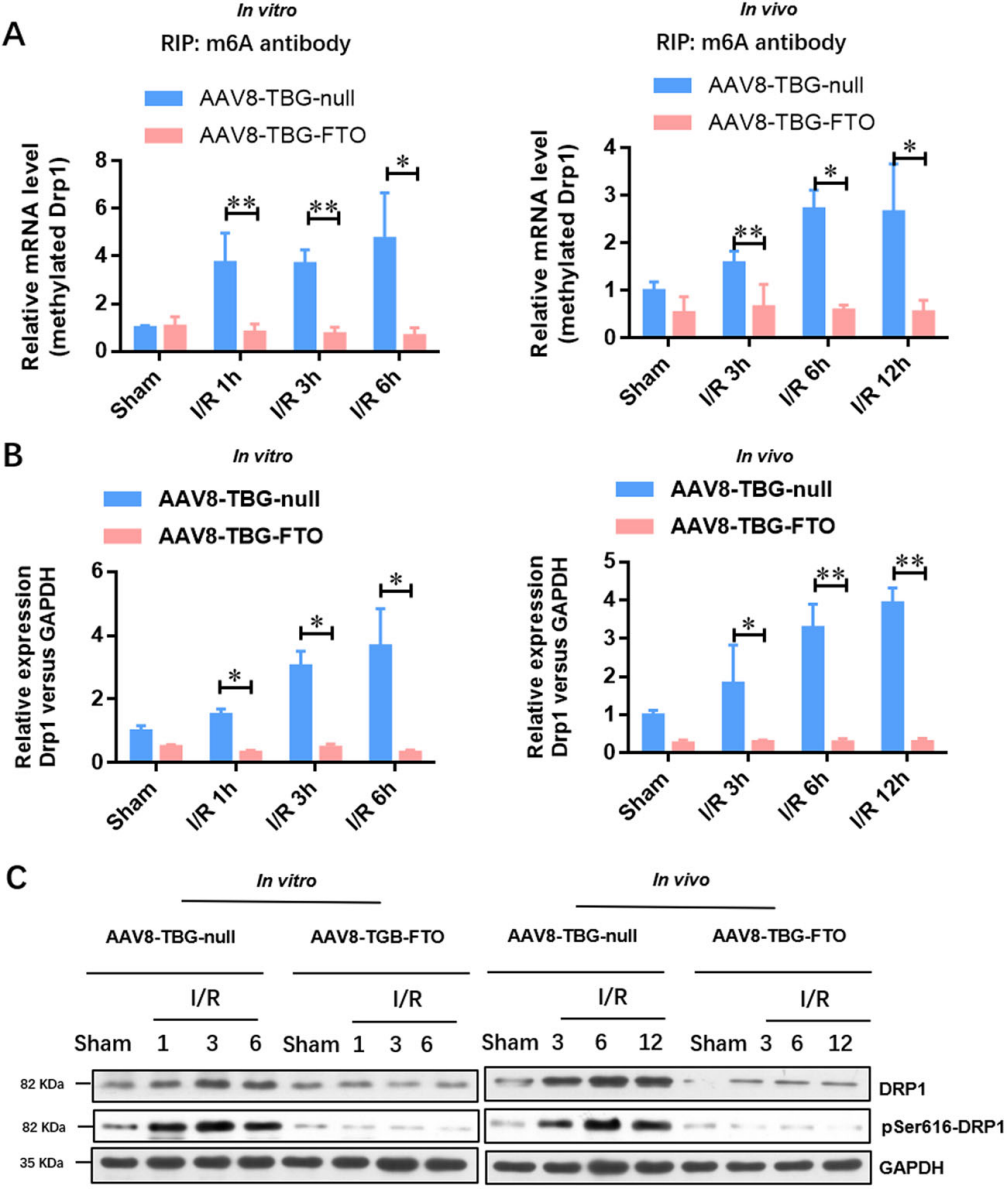
Liver-specific overexpression of FTO suppresses the IRI-elevated expression of Drp1 in hepatocytes upon IRI in vivo and in vitro. **A** Me-RIP qRT-PCR analysis was performed to evaluate the methylated Drp1 RNA level in the hepatocytes and hepatic tissues infected with control AAV8-TBG-null and AAV8-TBG-FTO during IRI. **B** qRT-PCR was adopted to analyze the expression of Drp1 at the mRNA level in the hepatocytes and hepatic tissues infected with control AAV8-TBG-null and AAV8-TBG-FTO during IRI. **C** Western blotting analysis was used to verify the expressions of total and activated Drp1 (pSer616) at the protein level in the hepatocytes and hepatic tissues infected with control AAV8-TBG-null and AAV8-TBG-FTO during IRI. *N* = 3; *P < 0.05 and **P < 0.01 vs. AAV8-TBG-NC group.

As Drp1 activation is critical for mitochondrial fragmentation, we further investigated whether FTO overexpression regulated the m6A methylation of Drp1 mRNA. Firstly, we predicted the potential methylation sites (Supplementary Fig. 1A) by a sequence-based m6A modification site predictor (SRAMPA, http://www.cuilab.cn/sramp/). Based on this prediction, we designed five pairs of specific primers for Drp1 mRNA (Supplementary Fig. 1B) and validated that the m6A methylation region of Drp1 mRNA in normal hepatocytes relied on very high confidence m6A site (Supplementary Fig. 1C, 2086 bp, AGACU). Figure 5A shows that the level of methylated Drp1 mRNA was elevated in liver and hepatocytes upon IRI, whereas it was suppressed in FTO-overexpressing hepatic tissues and hepatocytes. Furthermore, the expression of Drp1 at the mRNA level was increased in hepatocytes (in vitro) and hepatic tissues (in vivo), whereas it was suppressed by FTO overexpression (Fig. 5B). The expressions of total and activated Drp1 (pSer616) at the protein level in the liver upon IRI were further confirmed by Western blotting analysis (Fig. 5C). These results suggested that Drp1 was a potential downstream target of FTO in the progression of HIRI.

### Drp1 is a downstream target of FTO in the progression of HIRI

**Fig. 6:**
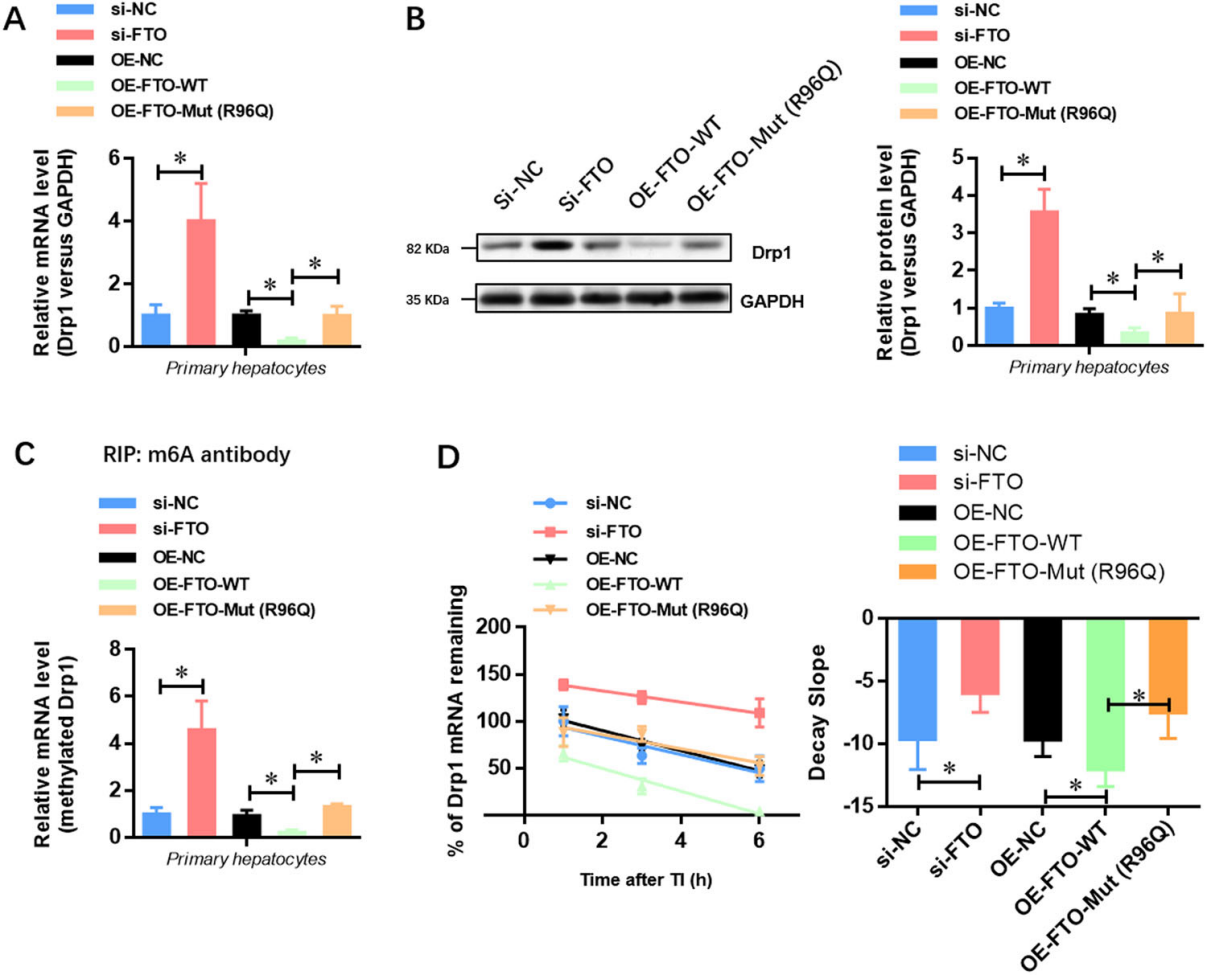
Drp1 is a downstream target of FTO in the progression of HIRI. **A** qRT-PCR analysis showed that overexpression of wild-type but not demethylase- mutated FTO decreased the Drp1 mRNA level in isolated hepatocytes. **B** Western blotting analysis showed that overexpression of wild-type but not demethylase-mutated FTO decreased the Drp1 protein level in isolated hepatocytes. **C** Me-RIP qRT-PCR analysis showed that overexpression of wild-type but not demethylase-mutated FTO decreased the methylated Drp1 level in isolated hepatocytes. **D** The curve and statistical analysis of Drp1 mRNA decay slope on the transfection of FTO siRNA, FTO-WT, FTO-Mut (R96Q), or the negative control after transcriptional inhibition (TI) were shown. *N* = 3; *P < 0.05 versus indicated group.

To further illustrate the molecular downstream of FTO and identify whether Drp1 was a direct target of FTO in the progression of HIRI, we generated an R96Q-mutated FTO construct with disordered enzymatic activity as previously described. The results showed that the expression of Drp1 at the mRNA (Fig. 6A) and protein levels (Fig. 6B) was elevated by FTO depletion, while it was suppressed by the overexpression of wild-type FTO. However, the expression of Drp1 was not affected when the mutated FTO (R96Q) was overexpressed in primary hepatocytes (Fig. 6A, B). Meanwhile, the level of methylated Drp1 mRNA was increased by FTO depletion, whereas overexpression of wild-type FTO but not mutated FTO decreased the level of methylated Drp1 mRNA (Fig. 6C). Similarly, after transcriptional inhibition, the mRNA decay rate of Drp1 was increased, while it was not affected by overexpression of mutated FTO (Fig. 6D). Taken together, these findings suggested that FTO repressed the expression of Drp1 through its m6A demethylase activity.

### Drp1 is critical for the protective effect of FTO against HIRI

**Fig. 7:**
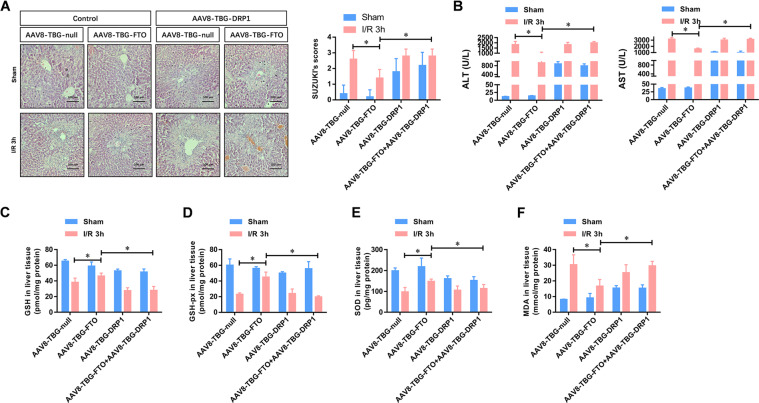
Drp1 contributes to the protective effect of FTO against HIRI. **A** Representative histopathologic images of liver sections harvested at 24 h after reperfusion (H&E staining; scale bars, 100 μm). The damage degree was graded using Suzuki criteria. **B** Blood samples were collected to determine the ALT and AST levels (U/L). **C**–**F** Tissue levels of GSH, GSH-Px, SOD, and MDA were detected using ELISA at indicated time points after reperfusion. *N* = 5; *P < 0.05 vs. indicated group.

To investigate whether Drp1 down-regulation was critical for the liver protective effect triggered by FTO overexpression, we restored the expression of Drp1 with adeno-associated virus-mediated liver-specific overexpression of Drp1 (AAV8-TBG-Drp1). Figure 7A shows that overexpression of Drp1 induced a larger area of necrosis along with sinusoidal congestion and cell swelling, which was even found in the sham group. Moreover, the reduced kidney histopathological lesions in FTO-overexpressing hepatic tissues were disappeared by Drp1 overexpression. Importantly, the reduced Suzuki criterion scores in FTO-overexpressing hepatic tissues were also significantly increased again by Drp1 overexpression (Fig. 7A). The attenuated levels of serum ALT and AST in AAV8-TBG-FTO mice at 3, 6, and 12 h after IRI were also markedly elevated by Drp1 overexpression (Fig. 7B). Moreover, the preserved levels of GSH, GSHPx, SOD, and MDA in AAV8-TBG-FTO hepatic tissues were reversed by Drp1 overexpression (Fig. 7C–F). These data collectively demonstrated that Drp1 overexpression effectively abrogated the protective effect of FTO against IRI.

## Discussion

The m6A mRNA methylation plays an important role in the recovery and regeneration of variant cells after IRI^12–14^. However, only very few studies have reported whether m6A methylation contributes to cell survival and recovery during HIRI. As the first m6A demethylase, FTO catalyzes m6A demethylation in a ferrous iron-dependent manner^26,27^. However, the roles of FTO in the progression of HIRI remain largely unexplored. In the present study, we found that FTO attenuated HIRI via inhibiting Drp1-mediated mitochondrial fragmentation. Our findings initially unveiled the role of FTO in the regulation of oxidative stress and the mitochondrial fragmentation of hepatocytes during IRI. Theoretically, it suggested that FTO was a potential therapeutic target for liver transplantation.

Here, we found that the expression of m6A demethylase FTO was decreased in hepatocytes (in vitro) and hepatic tissues (in vivo) after IRI, while the level of m6A methylated RNA was elevated during IRI. Functionally, we demonstrated that the elevated m6A methylated RNA level and FTO suppression contributed to the progression of IRI in hepatocytes. Moreover, liver-specific overexpression of FTO protected the hepatocytes against IRI-induced oxidative stress and mitochondrial fragmentation. This effect was further confirmed using the IRI model in FTO-overexpressing hepatocytes. Therefore, these data collectively suggested a protective role of FTO in hepatocytes upon IRI.

In the present study, we illustrated that Drp1 was a downstream target of FTO in the progression of HIRI. The morphology, distribution, and function of mitochondria are sustained by the mitochondrial fission and fusion hemostasis. Once the balance between these processes is disturbed, cell or organ dysfunction and abnormal mitochondrial redistribution will result in several diseases^28^. Drp1 plays a critical role in cell survival through mediating the mitochondrial fission process^29^ and regulating both cellular and organ dynamics, such as apoptosis, acute organ injury, development, and so on^30^. In the present study, we illustrated that FTO repressed the expression of Drp1 through its m6A demethylase activity. The methylated mRNA level of Drp1 was reduced in both FTO-overexpressing hepatic tissues and isolated primary hepatocytes. Our study further suggested that overexpression of wild-type FTO, but not mutant FTO, decreased the levels of methylated Drp1 mRNA and translated Drp1 protein. Finally, adeno-associated virus-mediated liver-specific overexpression of Drp1 effectively abolished the protective effect of FTO against IRI.

Collectively, our findings demonstrated that the m6A demethylase FTO ameliorated HIRI via inhibiting Drp1-mediated mitochondrial fragmentation. These results provided valuable insights into the understanding of HIRI and helped reveal new therapeutic targets for liver transplantation.

## Supplementary information

Supplementary Figure 1

Supplementary Figure 2

Supplementary Figure Legends
