# Supplementary figures and images for: N6-methyladenosine demethylase FTO impairs hepatic ischemia–reperfusion injury via inhibiting Drp1-mediated mitochondrial fragmentation
Source: Cell Death Dis. 2021 May 4;12(5):442. doi: 10.1038/s41419-021-03622-x (PMC8096847; doi:10.1038/s41419-021-03622-x)

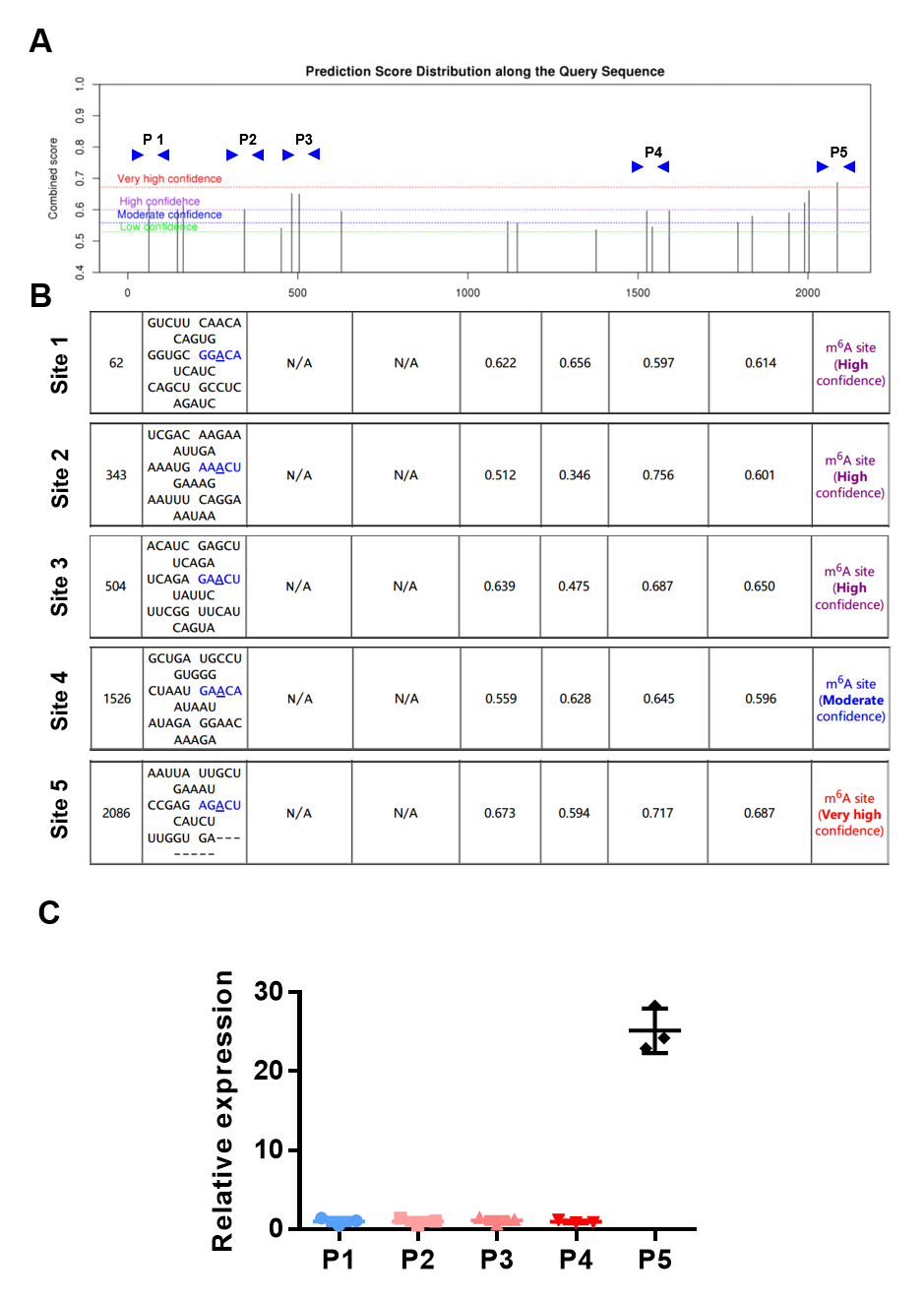

Supplement: Supplementary file 1 — Supplementary Figure 1 [file 41419_2021_3622_MOESM1_ESM.tif]

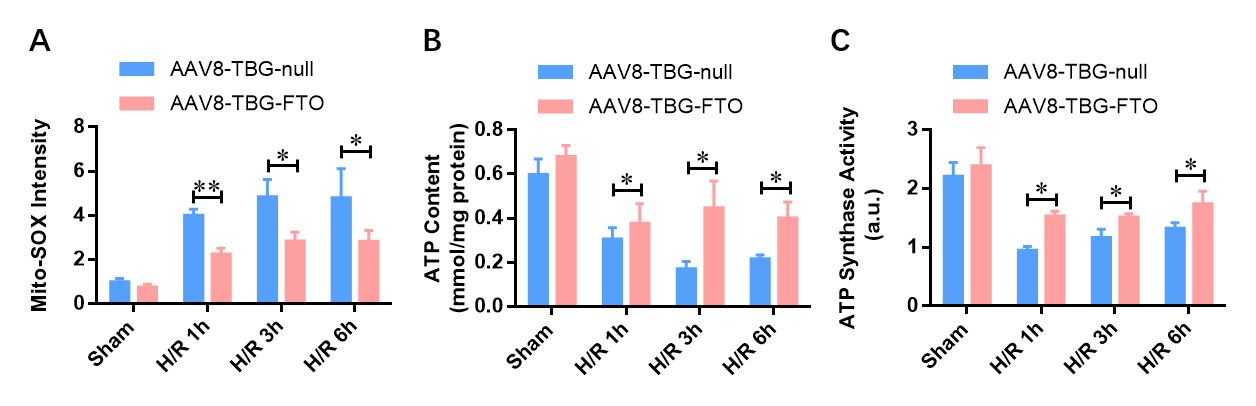

Supplement: Supplementary file 2 — Supplementary Figure 2 [file 41419_2021_3622_MOESM2_ESM.tif]
