# Supplementary material for: N6-methyladenosine demethylase FTO impairs hepatic ischemia–reperfusion injury via inhibiting Drp1-mediated mitochondrial fragmentation
Source: Cell Death Dis. 2021 May 4;12(5):442. doi: 10.1038/s41419-021-03622-x (PMC8096847; doi:10.1038/s41419-021-03622-x)
Supplement: Supplementary file 3 — Supplementary Figure Legends [file 41419_2021_3622_MOESM3_ESM.docx]

# N6-methyladenosine demethylase FTO impairs hepatic ischemia-reperfusion injury via inhibiting Drp1-mediated mitochondrial fragmentation

Running title: FTO impairs hepatic ischemia-reperfusion injury

Ying Dong Du^1#^, Wen Yuan Guo^2#^, Cong Hui Han^3#^, Ying Wang^1^, Xiao Song Chen^4^, Jin Long Liu^5*^, Nan Zhu^6*^, Xin Wang^7*^

1 Department of Transplantation and Hepatic Surgery, 970 Hospital of the PLA Joint Logistic Support Force, Yantai,

2 Department of Liver Surgery and Organ Transplantation, Changzheng Hospital, Naval Medical University

3 Department of Urology, The Affiliated School of Clinical Medicine of Xuzhou Medical College, Xuzhou Central Hospital, Xuzhou

4 Department of Transplantation and Hepatic Surgery, Ren Ji Hospital, Shanghai Jiaotong University School of Medicine, Shanghai, China;

5 Department of Biotechnology and Pathology, School of Medical Technology, Shanghai University of Medicine & Health Sciences, Shanghai

6 The Fifth Hepatic Surgery Department, Shanghai Eastern Hepatobiliary Surgery Hospital, Shanghai

7 Department of Hepatic Surgery, Zhongshan Hospital, Fudan University, Shanghai

^#^Theses authors contributed equally to this work.

*Correspondence to:

Jin Long Liu, liujl@sumhs.edu.cn, Department of Biotechnology and Pathology, School of Medical Technology, Shanghai University of Medicine & Health Sciences, 279 Zhouzhu Highway, Shanghai, 201318, China

Nan Zhu, drnanzhu@163.com, the Fifth Hepatic Surgery Department, Shanghai Eastern Hepatobiliary Surgery Hospital, No. 225 Changhai Road Yangpu District, Shanghai 200438, China

Xin Wang, drwangxin@163.com, Department of Hepatic Surgery, Zhongshan Hospital, Fudan University, No.180 Fenglin Road, Shanghai, 200032, China

**Supplementary Figure Legends**

**Supplementary Figure 1.** the m6A methylation region of Drp1 mRNA was relied on a very high confidence m6A site (supplementary Figure 1C, 2086bp, AGACU). (A). the potential methylation sites (supplementary Figure 1A) by a sequence-based N6-methyladenosine (m6A) modification site predictor (SRAMPA, <http://www.cuilab.cn/sramp/>); (B). Based on this prediction, we designed 5 pairs specific primer for Drp1 Mrna;(C). The m6A methylation region of Drp1 mRNA was verified to be on a very high confidence m6A site (2086bp, AGACU) by real-time PCR.

**Supplementary Figure 2.** **FTO overexpression alleviates the IRI impaired mitochondrial function.** (A) Mitochondrial ROS production in the hepatocytes infected with control AAV8-TBG-null and AAV8-TBG-FTO during H/R was detected by Mito-SOX assays. (B) The total level of ATP content in hepatocytes was detected by ATP Quantification Kit. (C) The ATP synthase activity in hepatocytes was detected by ATP synthase activity. N=3; **P*<0.05 and ***P*<0.01 versus AAV8-TBG-NC group.
